# Cell-Free DNA From Metastatic Pancreatic Neuroendocrine Tumor Patients Contains Tumor-Specific Mutations and Copy Number Variations

**DOI:** 10.3389/fonc.2018.00467

**Published:** 2018-11-01

**Authors:** Gitta Boons, Timon Vandamme, Marc Peeters, Matthias Beyens, Ann Driessen, Katrien Janssens, Karen Zwaenepoel, Geert Roeyen, Guy Van Camp, Ken Op de Beeck

**Affiliations:** ^1^Center for Oncological Research, University of Antwerp, Antwerp, Belgium; ^2^Center of Medical Genetics Antwerp, Antwerp University Hospital, University of Antwerp, Edegem, Belgium; ^3^Department of Oncology, Antwerp University Hospital, University of Antwerp, Edegem, Belgium; ^4^Section of Endocrinology, Department of Internal Medicine, Erasmus Medical Center, Rotterdam, Netherlands; ^5^Department of Pathology, Antwerp University Hospital, University of Antwerp, Edegem, Belgium; ^6^Department of Hepatobiliary, Endocrine and Transplantation Surgery, Antwerp University Hospital, University of Antwerp, Edegem, Belgium

**Keywords:** pancreatic neuroendocrine tumors, circulating tumor DNA, cell-free DNA, biomarkers, droplet digital PCR, whole-exome sequencing, shallow whole-genome sequencing

## Abstract

**Background:** Detection of tumor-specific alterations in cell-free DNA (cfDNA) has proven valuable as a liquid biopsy for several types of cancer. So far, use of cfDNA remains unexplored for pancreatic neuroendocrine tumor (PNET) patients.

**Methods:** From 10 PNET patients, fresh frozen tumor tissue, buffy coat and plasma samples were collected. Whole-exome sequencing of primary tumor and germline DNA was performed to identify tumor-specific variants and copy number variations (CNVs). Subsequently, tumor-specific variants were quantified in plasma cfDNA with droplet digital PCR. In addition, CNV analysis of cfDNA was performed using shallow whole-genome sequencing.

**Results:** Tumor-specific variants were detected in perioperative plasma samples of two PNET patients, at variant allele fractions (VAFs) of respectively 19 and 21%. Both patients had metastatic disease at time of surgery, while the other patients presented with localized disease. In the metastatic patients, CNV profiles of tumor tissue and cfDNA were significantly correlated. A follow-up plasma sample of a metastatic patient demonstrated an increased VAF (57%) and an increased chromosomal instability, in parallel with an increase in tumor burden.

**Conclusions:** We are the first to report the presence of tumor-specific genetic alterations in cfDNA of metastatic PNET patients and their evolution during disease progression. Additionally, CNV analysis in cfDNA shows potential as a liquid biopsy.

## Introduction

Pancreatic neuroendocrine tumors (PNETs) are rare tumors with an incidence rate of 0.48 per 100,000 according to the Surveillance, Epidemiology, and End Results (SEER) program (1). Surgical resection of a PNET is often curative in early-stage disease, but 50% of cases present with unresectable disease at time of diagnosis (2). Patient diagnosis, follow-up and treatment are based on imaging, tumor (re)biopsies and biomarker assessment. Taking a biopsy is associated with potential complications and is therefore not feasible in some cases. Currently, Chromogranin A is the most widely used circulating biomarker in PNETs, but its diagnostic sensitivity and specificity are low. In addition, recent reports show limited value for Chromogranin A as a follow-up marker (3). Hence, new biomarkers are needed (4). Circulating tumor DNA (ctDNA) is the proportion of cell-free DNA (cfDNA) in the blood plasma that is released by a tumor as a result of apoptosis, necrosis and active secretion (5). The ctDNA can be detected and quantified in cfDNA through tumor-specific genetic alterations. ctDNA has been extensively studied in cancer patients as an alternative for tissue biopsies and for its biomarker potential in different stages of disease, as summarized by Wan et al. (6). In PNETs, however, ctDNA remains largely unexplored. This study aimed to demonstrate the presence of ctDNA in PNET patients through the detection of both tumor-specific point mutations and copy number variations (CNVs) using droplet digital PCR (ddPCR) and shallow whole-genome sequencing (sWGS), respectively.

## Materials and methods

### Patients

Ten patients diagnosed with a sporadic PNET and undergoing surgery for their primary tumor at the Antwerp University Hospital (UZA) were prospectively included in this study. Eight patients presented with limited, localized disease, while two patients had metastatic disease at time of surgery. All patients underwent surgery with curative intent. Since patient no. 3 presented with metastatic WHO2010 grade 3 disease (Supplementary Figure 1), he first started cisplatin-etoposide treatment. Only after showing a sustained partial response after 6 cycles, the decision was made to perform debulking surgery with curative intent. The other metastatic patient (no. 7; Supplementary Figure 2) was planned to undergo a two-stage-procedure, first a pancreatectomy with lymph node clearance and in a later moment, a liver transplantation to clear liver metastases. However, disease recurred before transplantation could be performed. In all patients, fresh frozen tumor tissue from pancreatic resection, perioperative blood samples in EDTA tubes and clinicopathological data were collected with informed consent. From case 7, an additional blood sample was taken during follow-up, 23 months after surgery and 12 days before succumbing to his disease. After a median follow-up time of 20 months (range: 11–31 months), seven patients were alive and disease-free, while patients 3 and 7 died due to their disease. One patient was lost to follow-up. The human biological material was provided by Biobank@UZA (Antwerp, Belgium; ID:BE71030031000)^1^ and the study was approved by the local ethics committee (Antwerp University Hospital/University of Antwerp).

### DNA extraction

DNA was isolated from primary tumor tissue (tumor DNA), buffy coat (germline DNA) and plasma (cfDNA) using the AllPrep DNA/RNA Micro kit (Qiagen, Hilden, Germany), QIAamp DNA Blood Mini kit (Qiagen) and the Maxwell RSC ccfDNA Plasma Kit for large volumes (Promega, Madison, WI, USA), respectively. DNA concentrations were assessed using Qubit 2.0 fluorometer (Thermo Fisher Scientific, Eugene, OR, USA).

### Whole-exome sequencing to detect tumor-specific alterations

Tumor and germline DNA were subjected to whole-exome sequencing (WES), using hybridization-based target enrichment with NimbleGen SeqCap EZ Human Exome v3.0 (Roche, Basel, Switzerland), on an Illumina NextSeq500 platform (Illumina, San Diego, CA, USA). Further analysis was performed using in-house analysis pipelines and paired variant callers VarScan2 (v2.4.2) (7) and MuTect2 (v1.1.5) (8) were used to call tumor-specific variants. Because WES data analysis provides multiple tumor-specific variants per patient, variant filtering in VariantDB (9) and prioritization were performed to select one target per patient for ddPCR. First, only rare non-synonymous single nucleotide variants (minor allele frequency <0.01 in dbSNP v142 10, ExAC v03 11 and 1000Genomes april2012 12) were identified. Then, alterations with a variant allele fraction (VAF) lower than 20% were excluded to allow validation of tumor-specific state using Sanger sequencing. Next, variants were prioritized that lie in previously described neuroendocrine tumor-associated genes, in COSMIC v82 cancer census genes (13) or variants with a high predicted pathogenicity by CADD PHRED (14) and SIFT effect (15). Selected variants, one per patient, were validated using Sanger sequencing on the 3130xl Genetic Analyser (Applied Biosystems Inc., Foster City, CA, USA) platform.

For CNV analysis, we developed an in-house pipeline that employs an algorithm to divide the genome into non-overlapping 50 kb-bins and subsequently counts all mapped sequencing reads for each sample within each bin. Next, logR-ratios were calculated for every tumor/normal pair.

### Droplet digital PCR for single nucleotide variants

Custom-made, variant-specific primer/probe assays were ordered from Bio-Rad to perform genotyping of cfDNA on the QX200 Droplet Digital PCR System (Bio-Rad, CA, USA). Specific sequences of primers and probes are not disclosed by Bio-Rad. However, sequences containing the 60–100 bp-sized amplicons are given in Supplementary Table 1. In short, 20 μL reaction mixtures consisting of 10 μL Supermix for Probes (no dUTP; Bio-Rad), 1 μL ddPCR assay mix (Bio-Rad) and 9 μL DNA and nuclease-free water, were partitioned in approximately 20,000 nanoliter-droplets with the QX200 Droplet Generator. Droplets were transferred to a PCR plate and subjected to PCR amplification (95°C × 10 min, (94°C × 30 s, 55°C × 1 min) × 40, 98°C × 10 min, 4°C hold; ramp rate 2.5°C/s) followed by read-out. Tumor and germline DNA were used, respectively, as positive and negative control for the mutation. Additionally, template-negative reactions were run. Droplets were manually called as mutant-only, wild-type (WT)-only, double-positive or template-negative using the QuantaSoft software package v1.7.4 (Bio-Rad).

### Shallow whole-genome sequencing of cell-free DNA

10–20 ng of cfDNA was used as input for sWGS aiming for a coverage of 0.3-fold. Library preparation was performed using the Truseq Nano DNA HT library prep kit (Illumina) with dual-indexing and sequencing was performed on the NextSeq500 platform (Illumina). CNVs were detected by applying the R-package QDNAseq (16).

## Results

### Whole-exome sequencing and variant selection

**Figure 1:**
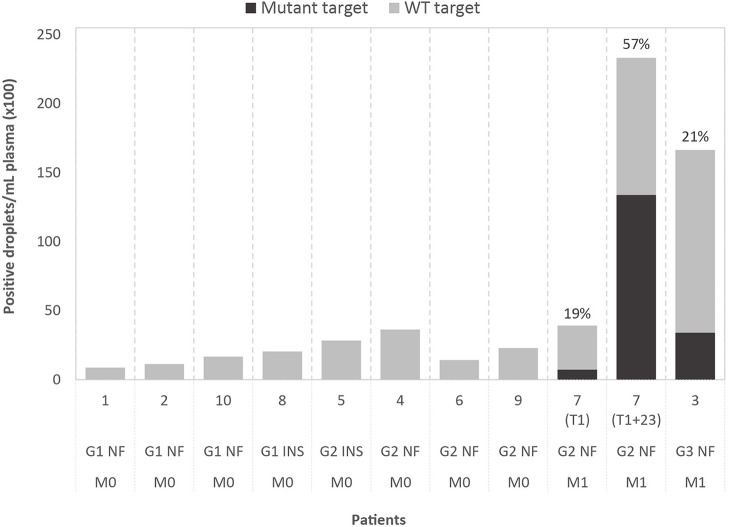
Results of droplet digital PCR (ddPCR) on cell-free DNA of ten patients, grouped by WHO2010 grade (G), functionality of the tumor (NF, non-functional; INS, insulinoma) and presence of metastasis (M0, no metastasis; M1, metastasis present). The graph shows the number of positive droplets per 1 mL of plasma, for both mutant (black) and wild-type (WT; gray) target. For mutant-positive patients, the variant allele fractions (VAFs) are indicated. The selected ddPCR targets are, from left to right, chr3:g.98251584T>A (*GPR15*), chr2:g.46603856C>T (*EPAS1*), chr21:g.39754856C>A (*ERG*), chr20:g.46288182C>T (*SULF2*), chr5:169477296C>T (*DOCK2*), chr2:g.111416130T>G (*BUB1*), chr2:g.204073466C>G (*NBEAL1*), chr16:g.15854467G>A (*MYH11*), chr11:g.64575561C>A (*MEN1*), chr6:g.33288573G>A (*DAXX*) (in GRCh37/hg19). For case 7, results obtained with the same ddPCR assay are shown for the perioperative plasma sample (T1) and the follow-up plasma sample (T1+23 months).

WES was performed on primary tumor tissue and corresponding germline samples with an average target base coverage of 108 ± 8-fold and 35 ± 7-fold, respectively. The goal of the WES analysis was to identify a tumor-specific variant for every patient which could then be detected in cfDNA of the corresponding patient. By applying the filters described in the methods section, we were able to select a single variant for every patient, which was validated with Sanger sequencing to confirm tumor-specificity (legend Figure 1). Our analysis revealed several interesting mutations in known PNET-associated genes, including missense mutations in *MEN1* and *EPAS1* and a stopgain mutation in *DAXX* (17, 18).

### Tumor-specific variants can be detected in cell-free DNA of metastatic patients

Custom ddPCR assays were designed for detection of the selected tumor-specific variants (mutant targets) with normal, WT targets as control. Analysis was performed on DNA extracted from tumor tissue, buffy coat and plasma. In tumor DNA, both mutant and WT targets could be detected by ddPCR, with VAFs showing a significant correlation with VAFs detected by WES (Pearson's *r* = 0.8786; *p* < 0.001). WT targets could be detected in cfDNA of all patients and two of the cases also tested positive for the tumor-specific mutation, with VAFs of respectively 19 and 21%. Droplet counts per mL plasma are shown in Figure 1. Assuming a limit for ctDNA-positivity of two mutant-positive droplets, our median detection limit based on the total amount of positive droplets is 0.27% (range: 0.06–0.63%). Remarkably, both patients that tested positive presented with metastatic disease before surgery, while the others presented with localized disease. The median plasma cfDNA concentration, estimated by Qubit, was 16 ng/mL (range: 4–30 ng/mL) for patients with localized disease, which is considerably lower than cfDNA concentrations in patients with metastatic disease (50 ng/mL and 81 ng/mL).

For case 7, two plasma samples were available, one perioperative (T1) and one follow-up sample, taken 23 months after surgery (T1+23 months). Plasma of both timepoints tested positive for the mutation, with an increase in VAF from 19 to 57% and in cfDNA concentration from 50 to 423 ng/mL, in line with the diffuse liver and bone invasion on T1+23 (Supplementary Figure 3).

### Reclassification of WHO grade 3 patient based on a liquid biopsy

Case no. 3 was diagnosed with metastatic WHO2010 grade 3 disease. In 2017, however, a new WHO grading system was implemented that distinguishes between well-differentiated grade 3 neuroendocrine tumors and poorly differentiated grade 3 neuroendocrine carcinomas. Tang et al. (19) described the most common molecular alterations associated with both types. In DNA extracted from both tumor tissue and plasma of our grade 3 case, we were able to detect a *DAXX* loss-of-function mutation, suggestive for classification as a well-differentiated grade 3 neuroendocrine tumor (19). To confirm our hypothesis based on molecular analysis, review by a dedicated pathologist was performed (Supplementary Figure 1). This showed indeed a morphologically well-differentiated PNET with a high Ki-67 (>20%). Remarkably, expression of the Ki-67 marker varied strongly across the tumor with hotspot regions reaching Ki-67 values as high as 66%, indicating tumor heterogeneity.

### CNVs detected in cfDNA and tumor tissue show a good correlation

**Figure 2:**
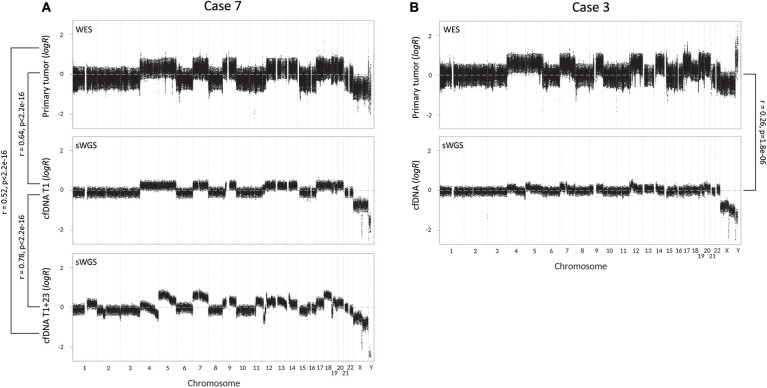
Copy number variation (CNV) profiles with correlations (Pearson's r) of tumor tissue and cell-free DNA (cfDNA) samples of the two metastatic cases. CNV profiles of tumor tissue and cfDNA were created respectively by whole-exome sequencing (WES) and shallow whole-genome sequencing (sWGS). **(A)** CNV profiles of case 7, with from top to bottom the CNV profile of tumor tissue, perioperative cfDNA sample (T1) and follow-up cfDNA sample, 23 months later (T1+23). **(B)** CNV profiles of case 3 (top: tumor tissue; bottom: cfDNA).

To further assess the biomarker potential of cfDNA, we constructed CNV profiles of cfDNA and primary tumor samples of our two metastatic cases (Figure 2). CNV profiles of primary tumor tissue and cfDNA(T1) of case 7 show a significant correlation (Pearson's *r* = 0.64, *p* < 2.2e^−16^). The CNV profile of the follow-up sample, cfDNA(T1+23), shows increased chromosomal instability, which is reflected by a lower Pearson's *r*-value than cfDNA(T1), when compared to the primary tumor (*r* = 0.52, *p* < 2.2e^−16^). The higher correlation between the two cfDNA samples (*r* = 0.78, *p* < 2.2e^−16^) can be explained by uniformity of the technique and the fact that sWGS creates more data points and, hence, a more stable CNV profile than WES. CNV profiles of primary tumor and perioperative cfDNA sample for case 3 are also significantly correlated (*r* = 0.26, *p* = 1.8e^−06^), but the correlation is less strong. In general, however, the same chromosomal regions seem to be affected in the tumor and the cfDNA sample, but logR ratios are closer to zero in cfDNA.

## Discussion

This study provides the first evidence for the presence of ctDNA in plasma of metastatic PNET patients, through ddPCR genotyping of cfDNA for tumor-specific variants. Tumor-specific variants were obtained for all patients through WES analysis of primary tumor tissue and germline DNA, but when genotyping variants in cfDNA of cases with localized disease, the variants could not be detected. This suggests that there is no ctDNA present or that a lower detection limit is required to detect it. Absence or presence of lower levels of ctDNA during early stage cancer have been described previously and the fact that PNETs are often indolent tumors, could also explain the absence of ctDNA in plasma (20). ctDNA-negative patients did not relapse during follow-up, while the two ctDNA-positive cases succumbed within 2 years after surgery to their disease, despite histology-confirmed R0 resection in case 3. Therefore, ctDNA analysis might help to differentiate between localized and metastatic disease, which has important prognostic and therapeutic implications, or help to detect relapse. This should be evaluated in further studies. Interestingly, we were able to detect a *DAXX* mutation in tumor tissue and plasma of a WHO2010 grade 3 patient (no. 3), which is suggestive for classification as a WHO2017 grade 3 well-differentiated neuroendocrine tumor, as opposed to a poorly differentiated neuroendocrine carcinoma (19). Pathology revision confirmed this diagnosis, showing potential for ctDNA to differentiate between the two types of WHO2017 grade 3 tumors, and possibly in the future also between other WHO grades as molecular research is ongoing (19, 21).

In both metastatic cases, a significant correlation was found between CNV profiles of tumor tissue and corresponding cfDNA, but there was a marked difference in the strength of the correlation. This might be explained by a difference in ctDNA fraction, if wrongly estimated by mutation analysis with ddPCR, or by tumor heterogeneity. Since central pathological review has demonstrated the presence of tumor heterogeneity in case 3, in which a weaker correlation was identified, tumor heterogeneity is the most likely explanation. CNV profiles are often characteristic for a certain tumor type (22). As many neuroendocrine tumors present with an unknown primary, CNV analysis of cfDNA to identify the primary tumor site might be a potential application.

Mutation and CNV analysis of a follow-up blood sample at progression showed an increase in cfDNA concentration, VAF and chromosomal instability. The increase in cfDNA concentration and VAF indicates an association with tumor burden, hinting toward a potential role for ctDNA as a follow-up marker for detection of treatment response or progression. The detection of an altered CNV profile, caused by disease progression and treatment pressure, means that cfDNA provides a real-time representation of cancer dynamics.

Our approach, where we first sequence the resected tumor followed by detection of tumor-specific variants in plasma, can only be applied for monitoring tumors in postoperative survey. However, other approaches might be explored in further studies, such as sequencing of tissue biopsies to identify tumor-specific variants, or detection of ctDNA by CNV analysis (as shown), methylation markers or sequencing of cfDNA (23, 24). In metastatic PNET cases, our results suggest that cfDNA might be a novel alternative to tissue biopsies for molecular profiling. Research on PNET tissue is being performed to identify prognostic and predictive genetic alterations, but few alterations have been validated so far (25). The possibility to detect genetic alterations in the blood instead of tissue would facilitate this research and future applicability due to easier access to tumor material in different stages of disease or treatment, evading the need for repeated tissue biopsies. Patients without treatment options could also benefit from ctDNA profiling through identification of actionable molecular alterations to allow inclusion in “molecular trials” with targeted therapies. Additionally, it is believed that all tumor cells release DNA, hence, molecular profiling of ctDNA creates a representation of alterations in the whole tumor, thereby evading the typical tumor heterogeneity problem of tissue biopsies.

To conclude, we report the first evidence for the presence of ctDNA in plasma of metastatic PNET patients and demonstrate its potential as a novel biomarker for PNETs. However, additional research on larger sample sizes and with multiple sampling timepoints per patient is required to further explore the possibilities of ctDNA in PNET patient management.

## Availability of data and materials

The datasets generated and analyzed during the current study are available from the corresponding author on reasonable request.

## Ethics statement

This study was carried out in accordance with the recommendations of the ethics committee of the Antwerp University Hospital/University of Antwerp with written informed consent from all subjects. All subjects gave written informed consent in accordance with the Declaration of Helsinki. The protocol was approved by the ethics committee of the Antwerp University Hospital/University of Antwerp (approval number 16/46/490).

## Author contributions

GB, TV, MP, GVC, and KOdB contributed to conception and design of the study and wrote the manuscript. GB, TV, and MB performed experiments and data analysis. GR and TV collected the samples. AD performed pathology review. All authors contributed to data interpretation and manuscript revision.

### Conflict of interest statement

The authors declare that the research was conducted in the absence of any commercial or financial relationships that could be construed as a potential conflict of interest.

## Footnotes

^1^*Biobank@UZA, BBMR-ERIC, Belgian Virtual Tumourbank funded by the National Cancer Plan; No. Access: (2), Last: April, 12, 2017. [BIORESOURCE]*.
