# Supplementary material for: Cell-Free DNA From Metastatic Pancreatic Neuroendocrine Tumor Patients Contains Tumor-Specific Mutations and Copy Number Variations
Source: Front Oncol. 2018 Nov 1;8:467. doi: 10.3389/fonc.2018.00467 (PMC6221938; doi:10.3389/fonc.2018.00467)
Supplement: Supplementary file 1 [file Data_Sheet_1.pdf]

Supplementary Material

Cell-free DNA from metastatic pancreatic neuroendocrine tumor patients contains tumor-specific mutations and copy number variations

Gitta Boons, Timon Vandamme, Marc Peeters, Matthias Beyens, Ann Driessen, Katrien Janssens, Karen Zwaenepoel, Geert Roeyen, Guy Van Camp, Ken Op de Beeck*

*** Correspondence:** Ken Op de Beeck: ken.opdebeeck@uantwerpen.be

1. Supplementary Figures

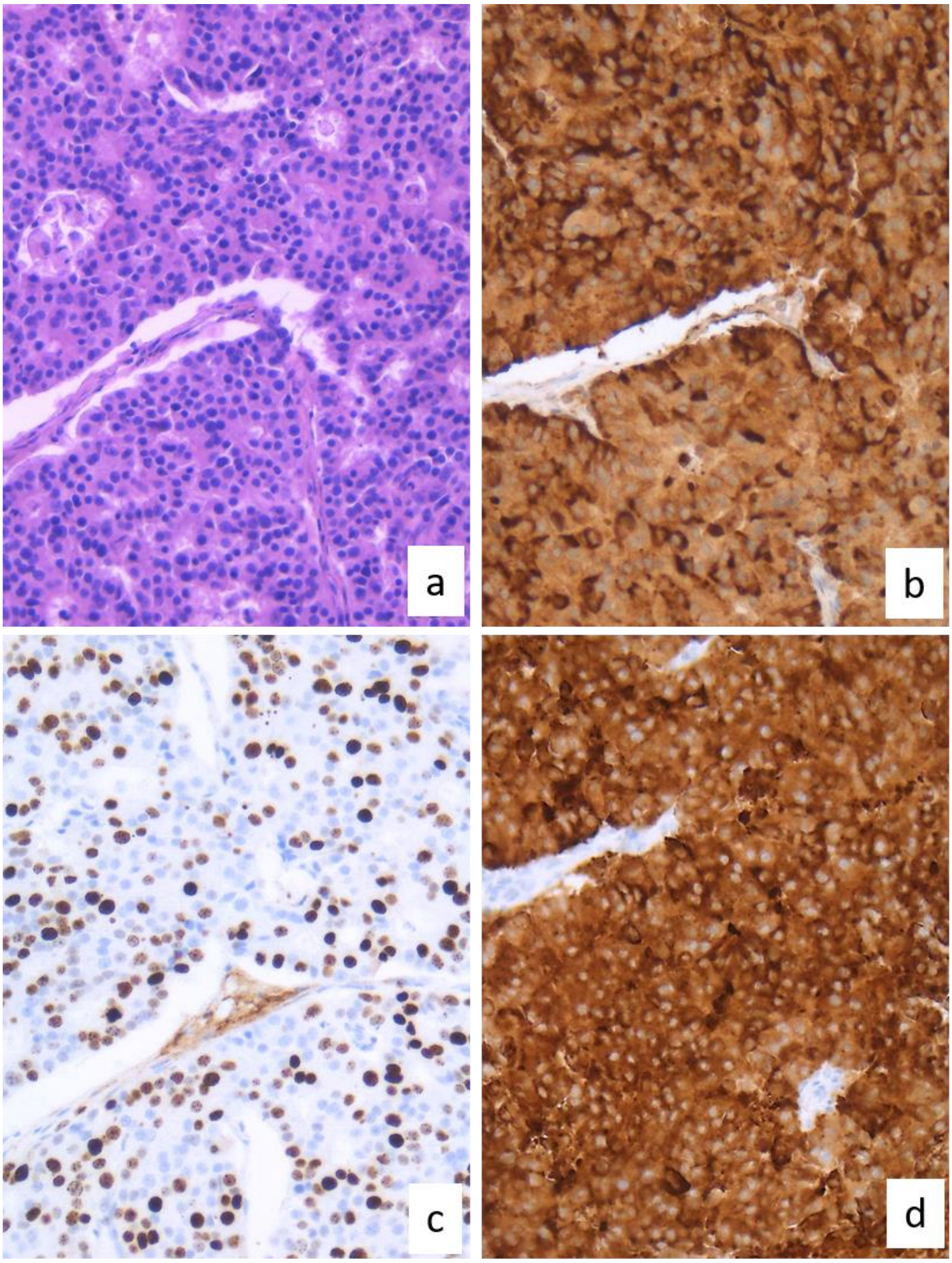

Supplementary Figure 1. Histologic images of case 3. This neuroendocrine tumor is characterized by a glandular growth pattern (a, HE, 20x) and is composed of tumor cells showing a strong expression of chromogranin (b, 20x) and synaptophysin (d, 20x). The tumor shows a high proliferation rate (c, Ki-67, 20x), corresponding to a well-differentiated grade 3 neuroendocrine tumor.

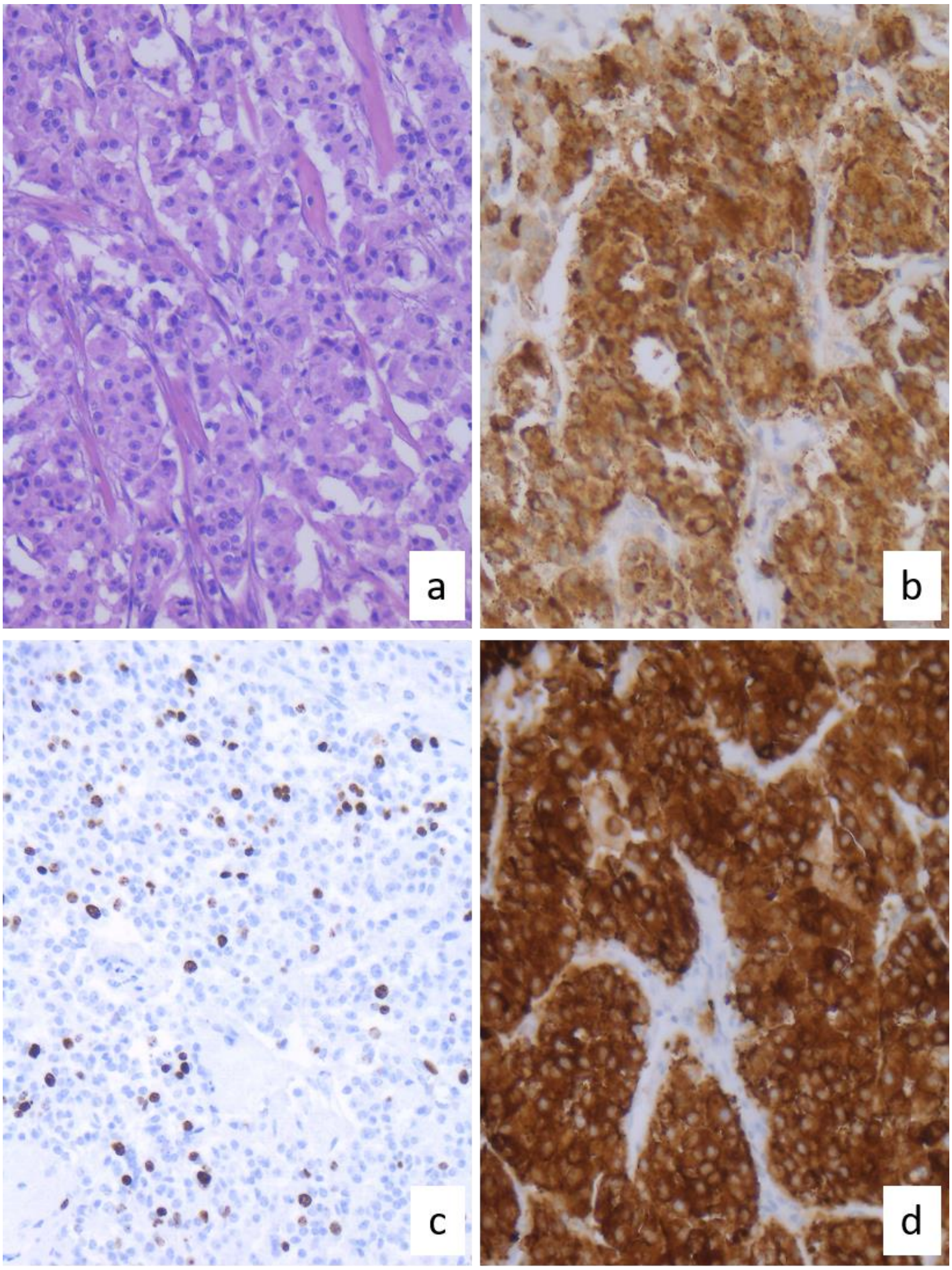

Supplementary Figure 2. Histologic images of case 7. This neuroendocrine tumor is characterized by a trabecular growth pattern (a, HE, 20x) and is composed of tumor cells showing a strong expression of chromogranin (b, 20x) and synaptophysin (d, 20x). The tumor shows a moderate proliferation rate (c, Ki-67, 20x), corresponding to a well-differentiated grade 2 neuroendocrine tumor.

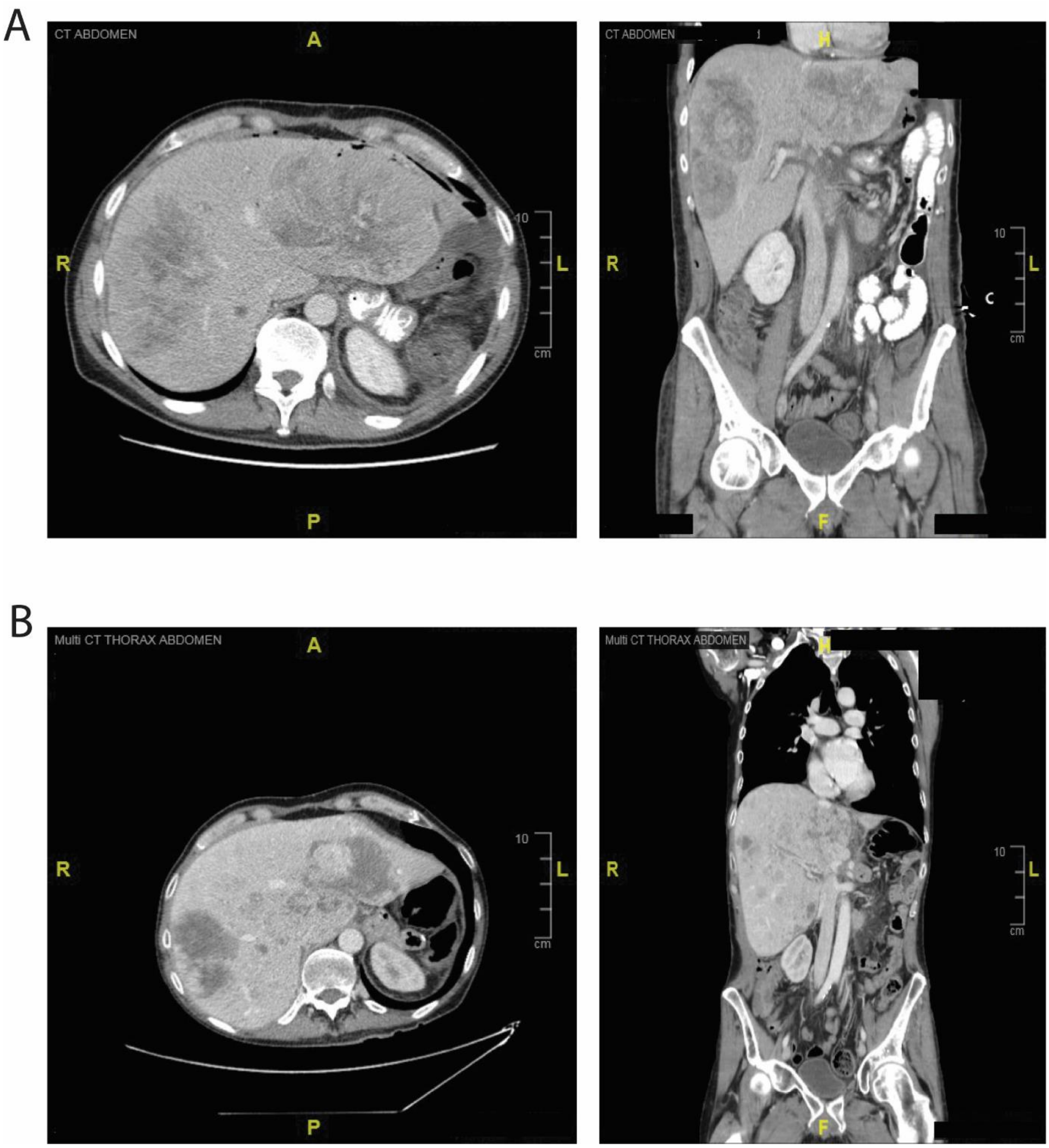

Supplementary Figure 3. Clinical images of patient 7 for both blood sampling timepoints, illustrating an increase in tumor burden. A) CT scan taken 1 week after the operation for the primary tumor, two large lesions are visible in the liver. B) CT scan taken 22 months after surgery and one month before the second plasma sampling. The two large liver lesions are still present, but in addition also diffuse liver metastasis are present. Furthermore, the patient also suffered from bone metastasis, which is not visible in these images.

2. Supplementary Tables

Supplementary Table 1. Overview of the selected variants for droplet digital PCR (ddPCR). For each variant the gene in which it is located, the chromosome location and the reference and alternative allele are shown. Furthermore, the amplicon size and amplicon-containing sequence of the assay for genotyping the variant, as received from Bio-Rad, are shown.

Patient	Gene	Chromosome location	Reference > Alternative allele	Amplicon size (bp)	Amplicon Sequence
1	*GPR15*	chr3:98251584	T>A	69	TGTTGCATTGCAAGGAAGCTGTGTGCCCATTACCAGCAATCAGGAAAGCACAACAAAAAGC[T/A]GAAGAAATCTATAAAGATCATCTTTATTGTCGTGGCAGCCTTTCTTGTCTCCTGGCTGCCC
2	*EPAS1*	chr2:46603856	C>T	65	GAAGAGTAACTTCCTATTCACCAAGCTAAAGGAGGAGCCCGAGGAGCTGGCCCAGCTGGCT[C/T]CCACCCCAGGAGACGCCATCATCTCTCTGGATTTCGGTGGGTGCTTCTTAGCTAAGCCAGG
3	*DAXX*	chr6:33288573	G>A	62	CCTGGCCTATAGTCATCTGTGAGGTGGCAGCCAAAGTTGTAGATGAGATCGAGGTGACGTC[G/A]CTCCTGTAACCTGATGCCCACATCTCGGAAGGCATCCTGAGCCATGAGCTGGAGCTGCTGT
4	*BUB1*	chr2:111416130	T>G	80	AGGAGCCTGAAACATATTCATGATGAAACCTTAAAGAACAAAAAGAATAATTGTAAACAGG[T/G]TGCAAAAGAGCTTTCATTAACTTGTCATCAGGTGCTACACACTCAGATAAAATACTTTACC
5	*DOCK2*	chr5:169477296	C>T	77	TCTAATGAGCATCCTTTCTCTGCAGAACAAAGTGTTCATCTACCGCGGGAAGGAATATGAG[C/T]GAAGAGAAGATTTCCAGATGCAGCTGATGACCCAGTTCCCCAATGCAGAGAAGATGAACAC
6	*NBEAL1*	chr2:204073466	C>G	67	TATGGACACACCAACGAGGTACTGAGTGTCGGCATCAGCACTGAGCTAGACATGGCAGTGT[C/G]AGGATCAAGGGTAAGATTTCACCTTTAAGAAATACTATTTATTTTGTCTACAAATTTATTA
7	*MEN1*	chr11:64575561	C>A	69	CATCCCGGAGACCCAGGGCCTGGCAGGCCCCAACCACAGCAAAGGCCACACCGGAGCTGTC[C/A]AATTTGGTGCCTGTGGAAGGGGGAGGTAATGAAAGAGGGTCCTCTGTGCTTTAACATGGGG
8	*SULF2*	chr20:46288182	C>T	70	GTAAGCTGGTTCCTCAACTCACAGTGATTTGGAAGAAGGTCTCTTCATTTCTGGCCACTTT[C/T]GACGCTGAAACTGCCTAAGTTTTCAAGTGTCAAAGGAGAATGAATGTTAACCATCACCAAG
9	*MYH11*	chr16:15854467	G>A	64	TGTCTGAGCTTTCTGTACCACATCTCGCCCAACCTTGATACGAGGAGTGAGGATGGATCTG[G/A]TGAAATCTGTCACATTAATTCCCATGAGGTGGCAAACTTTCTGAGCAGCTGGATGGAGAAA
10	*ERG*	chr21:39754856	C>A	96	ACATGCCATGCAGTTGCATATCAACGTCTGTTGATGGGCCACAGTCTCTCTCGTGTCTTTT[C/A]TCTTGTTTTTGATATGTTTCTATTTTTAAATACAGGTAGTTTTCCTTAAAATGGCATTATA
